# BMSC-Derived Exosomal miR-29a Promotes Angiogenesis and Osteogenesis

**DOI:** 10.3389/fcell.2020.608521

**Published:** 2020-12-09

**Authors:** Guo-dong Lu, Peng Cheng, Ting Liu, Zhong Wang

**Affiliations:** ^1^Department of Cardiology, The First Affiliated Hospital of Shihezi University Medical College, Shihezi, China; ^2^Division of Geriatric Endocrinology, The First Affiliated Hospital, Nanjing Medical University, Nanjing, China; ^3^Department of Endocrinology, Changsha Central Hospital, Changsha, China

**Keywords:** BMSCs-exosomes, miRNA-29a, angiogenesis, osteogenesis, osteoporosis

## Abstract

Angiogenesis and osteogenesis are tightly coupled during bone modeling and remodeling processes. Here we reported that bone marrow mesenchymal stem cell (BMSC)-derived exosomal miR-29a promotes angiogenesis and osteogenesis *in vitro* and *in vivo*. BMSC-derived exosomes (BMSCs-Exos) can be taken up by human umbilical vein endothelial cells (HUVECs) and promote the proliferation, migration, and tube formation of HUVECs. MiRNA-29a level was high in BMSCs-Exos and can be transported into HUVECs to regulate angiogenesis. VASH1 was identified as a direct target of miR-29a, mediating the effects of BMSC-derived exosomal miR-29a on angiogenesis. More interestingly, miR29a-loaded exosomes from engineered BMSCs (miR-29a-loaded BMSCs-Exos) showed a robust ability of promoting angiogenesis and osteogenesis *in vivo*. Taken together, these findings suggest that BMSC-derived exosomal miR-29a regulates angiogenesis and osteogenesis, and miR-29a-loaded BMSCs-Exos may serve as a potential therapeutic target for osteoporosis.

## Introduction

Bone undergoes sustained modeling and remodeling processes throughout life to ensure the integrity and structure of the skeleton (Wu et al., 2010). Angiogenesis is tightly coupled with bone formation and regeneration for proper bone homeostasis (Hui et al., 2014; Kusumbe et al., 2014; Yang et al., 2017). Recently, extracellular vesicles (EVs), such as exosomes and microvesicles, have been attracting a strong research interest for use as natural drug delivery systems (Chen et al., 2018; Tang and Wang, 2020). Mesenchymal stem cell (MSC)-derived exosomes have been reported to be involved in multiple physiology and pathology activities including osteogenesis, bone regeneration, osteoarthritis, cardiomyoblast hypoxia–reperfusion, cognitive impairment, inflammation, stroke, sepsis, and tumorigenesis (Zheng et al., 2018; Huang et al., 2020; Jin et al., 2020; Nakano et al., 2020; Tan et al., 2020; Venkat et al., 2020; Xu et al., 2020; Zou et al., 2020). Jia et al. (2020) report that exosomes secreted by young MSCs promote osteogenesis and bone formation in older rats. Liu et al. (2020) find that MSC-derived exosomes promote bone fracture healing by transferring miR-126. Zuo et al. (2019) report that bone marrow MSC (BMSC)-derived exosomes alleviate radiation-induced bone loss by activating Wnt/β-catenin signaling. However, the effects of BMSC-derived exosomes (BMSCs-Exos), especially the key microRNAs within the BMSCs-Exos, on angiogenesis and osteogenesis are not well studied.

MicroRNAs are a class of small non-coding RNAs of 19–22 nucleotides and involved in almost all cellular processes as a negative mediator of mRNA translational efficiency (Li et al., 2015). MicroRNAs post-transcriptionally decrease targeted gene expression through binding the sequence in the 3′ untranslated region (UTR) of the target mRNAs (Li et al., 2015). Recently, miRNAs were reported to be secreted and delivered into distal or local tissue and cells through circulation within exosomes (Hu et al., 2018; Vilaca-Faria et al., 2019; Song et al., 2020; Sun et al., 2020). Exosomes are EVs, with sizes arranged from 30 to 150 nm, released from multiple cell types. The functions of containing and transporting of bioactive protein, mRNAs, and miRNAs to other cells make exosomes a research hotspot of intracellular communications (Li and Tang, 2020). Recent studies report that exosomes from MSCs (Gong et al., 2017), smooth muscle cells (Zheng et al., 2017), tumor cells (Zhang et al., 2016), leukemia cells, and macrophages (Li M. et al., 2019) mediate angiogenesis. MSC-derived exosomes containing miR-30b, miR-30c, and miR-424 promote angiogenesis through targeting DLL4 (Ghosh et al., 2010; Bridge et al., 2012; Gong et al., 2017). Exosomal miR-301 derived from MSCs inhibits myocardial autophagy to protect myocardial infarction (Li Y. et al., 2019). MiR-155 within exosomes from smooth muscle cells induces endothelial injury and promotes atherosclerosis (Zheng et al., 2017). In gastric carcinoma, cell-derived microvesicles delivered miR-29a/c to suppress angiogenesis (Zhang et al., 2016). Li M. et al. (2019) report that macrophage-derived exosomes suppress the inflammation and promote the proliferation and migration of endothelial cells during wound healing. Li’s group reported that human induced pluripotent stem cell-derived exosomes promote bone defect repair through enhancing angiogenesis and osteogenesis in ovariectomy-induced osteoporotic rat model (Qi et al., 2016). However, the exact molecular mechanisms responsible for the effects of BMSCs-Exos on angiogenesis and osteogenesis remain elusive.

In this study, we reported that BMSCs-Exos can be transported into endothelial cells and promote the proliferation, migration, and tube formation of endothelial cells. MiRNA-29a level was high in BMSCs-Exos and promoted angiogenesis. VASH1 was identified as a direct target of miR-29a, mediating the effects of BMSC-derived exosomal miR-29a on angiogenesis. Moreover, miR-29a-loaded BMSCs-Exos showed a strong ability to promote angiogenesis and osteogenesis *in vivo*.

## Materials and Methods

### Animals and Treatment

Three-month-old male C57BL/6J mice were injected with miR-29a-loaded BMSCs-Exo, BMSCs-Exo, or phosphate-buffered saline (PBS) control *via* the tail vein (100 μg exosomes dissolved with 100 μl PBS or an equal volume of PBS) twice per week for 2 months. Then, the femurs were collected for analysis of angiogenesis and osteogenesis by immunostaining and micro-CT analysis. Animal care and experimental protocol were approved by the Animal Care and Use Committees of the Laboratory Animal Research Center at Shihezi University First Affiliated Hospital. The animals were maintained in a pathogen-free facility of the Laboratory Animal Research Center at Shihezi University First Affiliated Hospital, with free access to food and water prior to the initiation of experiments.

### BMSC-Derived Exosome Isolation and Characterization

We isolated BMSC exosomes based on the protocols described previously (Ying et al., 2017). Briefly, we dissected femora from C57BL/6J mice. Then, we crushed and digested the femurs with collagenase II at 37°C for 15 min to make a single-cell suspension. The BMSCs were FACS-sorted using (Sca-1^+^CD29^+^CD45^–^CD11b^–^) strategy and cultured in exosome-free medium for 72 h. We collected the medium of BMSCs and centrifuged such to remove dead cells or debris. The collected medium was subjected to ultracentrifugation at 100,000 *g* for 4-6 h at 4°C after filtration with a 0.22-μm filter. The exosome-containing pellet was washed and resuspended with PBS. We characterized the exosomes by detecting the expression of exosome-specific markers syntenin 1 and TSG101 by western blot, concentration by NanoSight analysis (Particle Metrix), and size distribution and morphology by transmission electron microscopy imaging (Hitachi H7500 TEM). For the *in vitro* experiments, exosomes (2 μg of exosomes per 1 × 10^5^ recipient cells) were added to the culture medium for 12 h, and then tube formation was measured.

### Tube Formation Assay

Fifty microliters of Matrigel (BD Biosciences) was plated in 96-well culture plates and then incubated at 37°C. Human umbilical vein endothelial cells (HUVECs) were cultured on Matrigel with different exosomes or other treatments. After incubation at 37°C for 4 h, tube formation was visualized under microscopy and analyzed by measuring the cumulative tube lengths.

### MTS Assay

The tetrazolium-dye-based MTS assay was used to evaluate cell growth rate as described previously (Zheng et al., 2017). In brief, 1 × 10^4^ cells per well were seeded in triplicate into 96-well plates for 24 h. Then, the cells were treated with different treatments according to design. After treatment, the cells were washed twice with PBS and treated with 2 mg/ml Hank’s buffer and incubated for 1 h. Then, the dark blue crystal should be seen under light microscopy. The crystals were dissolved in dimethyl sulfoxide, and the absorbance was measured with a Thermo Fluoroscan Ascent spectrometer at 570 nm.

### Wound-Healing Assay

A total of 5 × 10^5^ HUVECs were seeded in six-well plates. Scratch wounds were created using 100-ml sterile pipette tips when the cells grew to 95% confluence. The plates were washed twice with PBS to remove the suspended cells. Images were captured in three defined fields at 0 and 48 h, respectively.

### Immunoblotting

We performed western blotting analysis as previously described (Li et al., 2016b; Li C. et al., 2017). Exosome lysates were separated by SDS-PAGE blotted on polyvinylidene fluoride membranes (Millipore). The membranes were incubated with primary antibodies against syntenin 1(PA5-28826), TSG101 (ab125011), and VASH1 (EPR17420). All the primary antibodies were incubated at 4°C overnight, and specific proteins were visualized by enhanced chemiluminescence (ECL Kit; Amersham Biosciences).

### qRT-PCR Analysis

The total RNAs were extracted by Trizol (Thermo Fisher Scientific). Qiagen miRNeasy Mini Kit was used to extract exosomal miRNAs. cDNA was prepared using the SuperScript First-Strand Synthesis System (Invitrogen) for qRT-PCR analysis by SYBR GreenMaster Mix (Qiagen). 2-CT method was used to calculate the relative expression after normalization with GAPDH and U6 (Li et al., 2016a).

### Luciferase Activity Assay

Luciferase activity assays were conducted as reported previously (Yang et al., 2017). We constructed the wild-type and mutated VASH1 3′ UTR luciferase reporter plasmids with the potential binding site of miR-29a-3p and co-transfected them with miR-29a-3p mimics or miR-NC into HEK293 cells, respectively. Renilla luciferase reporter plasmids were used as internal control. The luciferase activities were measured by a Dual-Glo Luciferase Assay Kit (Promega).

### Quantification and Statistical Analysis

All data are presented as mean ± SEM. For comparisons between two groups, a two-tailed Student’s *t*-test was used. Comparisons between multiple groups were made using ANOVA, followed by Bonferroni post-test. All experiments were repeated at least three times, and representative experiments are shown. *p* < 0.05 was considered statistically significant.

## Results

### BMSC-Derived Exosomes Promoted Angiogenesis

**FIGURE 1:**
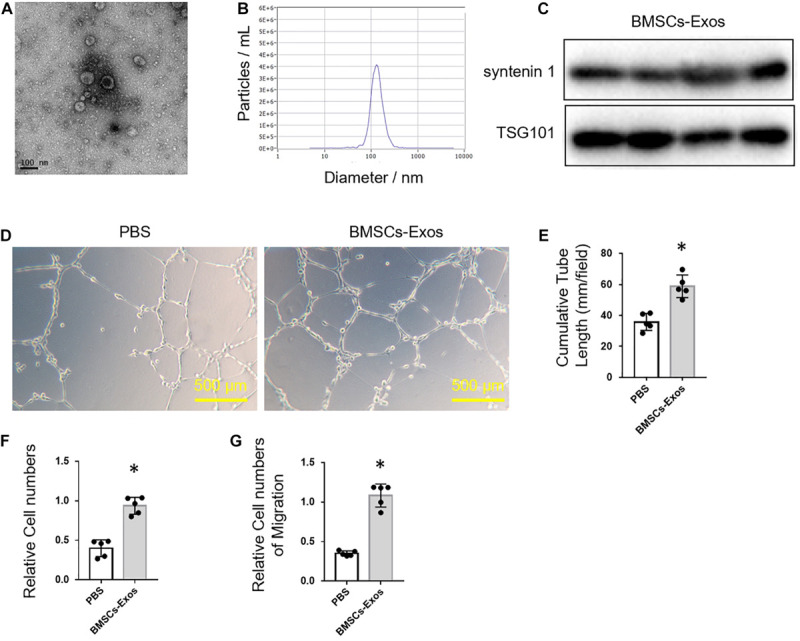
Bone marrow mesenchymal stem cell (BMSC)-derived exosomes promoted angiogenesis. **(A)** Representative picture of BMSC-derived exosomes collected by ultracentrifugation. Scale bar: 100 nm. **(B)** Size distribution profile of BMSC-derived exosomes. **(C)** Western blot analysis of exosome-specific markers, syntenin 1 and TSG101. Representative images of tube formation **(D)** and quantitative data of cumulative tube length **(E)** of human umbilical vein endothelial cell (HUVEC) cultured with phosphate-buffered saline control or BMSCs-Exos. Scale bars, 500 μm. **(F)** MTS assay was used to detect the proliferation of HUVECs. **(G)** Wound-healing assay was used to detect the migration of HUVECs; *N* = 5. Data are presented as mean ± SEM. *P < 0.05 as determined by Student’s *t*-test.

To obtain BMSCs-Exos, BMSCs were isolated from C57BL/6J mice by fluorescence-activated sorting (Sca-1^+^CD29^+^CD45^–^CD11b^–^) and then cultured with exosome-free medium for 72 h. The BMSCs-Exos were isolated by ultracentrifugation after the removal of dead cells and debris from the conditional medium. The exosomes were round-shaped vesicles with a bilayer membrane structure, as observed by electron microscopy (Figure 1A). The concentration of exosomes was around 1 to 2 × 10^10^ particles/ml, and the diameter predominantly ranged from 30 to 150 nm (Figure 1B). The exosome-specific protein markers (syntenin 1 and TSG 101) were highly expressed in these exosomes as evidenced by Western blotting analysis (Figure 1C).

Next, we tested the effects of BMSCs-Exos on angiogenesis. BMSCs-Exos and PBS control were added to the culture medium of HUVECs, respectively, for tube-like structure formation assay. The cumulative tube length was significantly longer in HUVECs treated with BMSCs-Exos compared to those treated with PBS control (Figures 1D,E). Furthermore, the proliferation and migration of BMSCs-Exos-treated HUVECs were dramatically increased compared to the PBS-treated control (Figures 1F,G). These data indicated that BMSCs-Exos promoted the proliferation and migration of endothelial cells and thus enhanced angiogenesis.

### BMSC-Derived Exosomal miR-29a Transported Into Endothelial Cells

**FIGURE 2:**
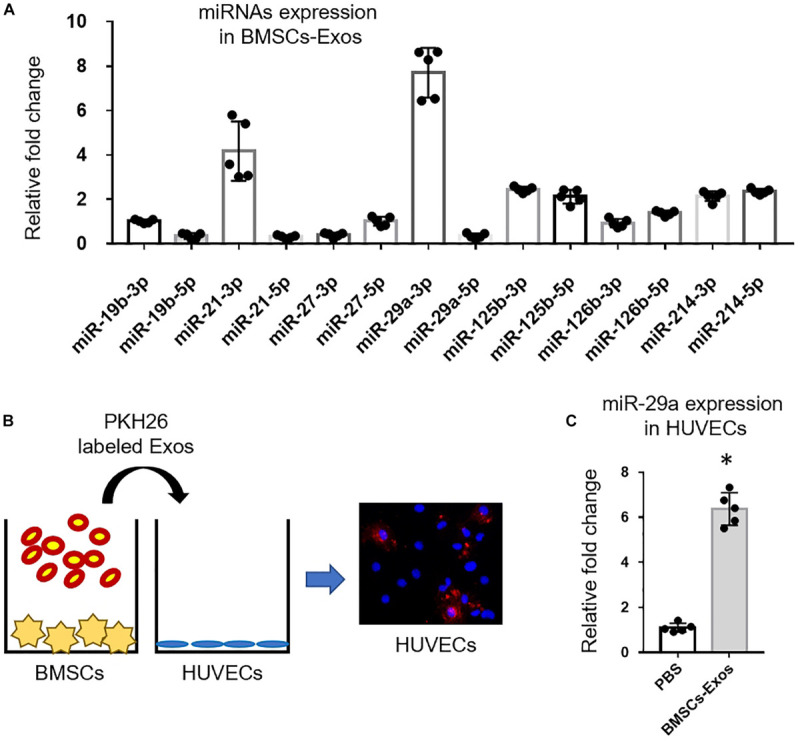
miR-29a was increased in bone marrow mesenchymal stem cells (BMSCs)-Exo and transported into endothelial cells. **(A)** qPCR analysis of angiogenesis-related miRNA expression in BMSCs. **(B)** Exos from BMSCs were labeled with PKH26 and then added to human umbilical vein endothelial cell (HUVEC) cultures. **(C)** After treatment of BMSCs-Exos for 24 h, the levels of miR-29a in HUVECs were measured by qPCR analysis. Data are presented as mean ± SEM; *N* = 5 per group. *p < 0.05, Student’s *t*-test.

Exosomes contain and transport bioactive proteins, mRNAs, and microRNAs into distal and nearby cells. Next, we measured the angiogenesis-related miRNA (miR-19b, miR-21, miR-27, miR-29a, miR-125b, miR-126b, and miR-214) expression in BMSCs-Exos. A qPCR analysis revealed that miR-29a-3p, but not miR-29a-5p, was the most highly expressed microRNA among the detected miRNAs within BMSCs-Exos (Figure 2A). Thus, we chose miR-29a-3p to study. Our previous study found that exosomal miR-29a could be taken up by adipocytes, myocytes, and hepatocytes and then regulate insulin resistance (Liu T. et al., 2019). In this study, we tested whether exosomal miR-29a could be transferred into endothelial cells to affect angiogenesis. First, we tested whether BMSCs-Exos can be transferred into endothelial cells. The BMSCs-Exos were labeled with the fluorescent dye PKH26 and then added into the culture medium of HUVECs. The red fluorescence staining showed that HUVECs exhibited an efficient uptake of BMSCs-Exos (Figure 2B). Furthermore, after culturing with BMSCs-Exos, the miR-29a level in these recipient HUVECs was significantly higher than those cultured with PBS control (Figure 2C).

### BMSCs-Exosomal miR-29a Promoted Angiogenesis

**FIGURE 3:**
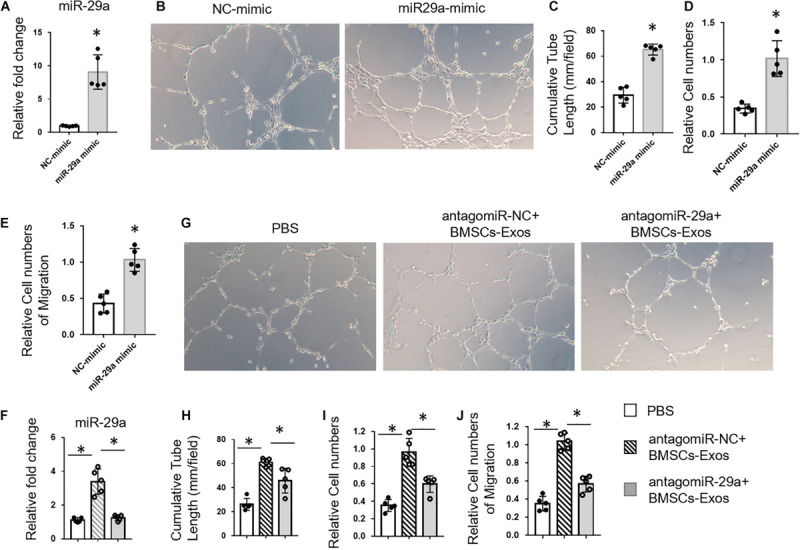
Bone marrow mesenchymal stem cells (BMSCs)-exosomal miR-29a promoted angiogenesis. **(A)** qPCR analysis of miR-29a expression in human umbilical vein endothelial cells (HUVECs) transfected with miR-29a mimic. Representative images of tube formation **(B)** and quantitative data of cumulative tube length **(C)** of HUVECs transfected with miR-29a or control mimics. **(D)** MTS assay was used to detect the proliferation of HUVECs. **(E)** Wound-healing assay was used to detect the migration of HUVECs. **(F)** qPCR analysis of miR-29a expression in exosomes collected from BMSCs with transfection of antagomir-29a. Representative images of tube formation **(G)** and quantitative data of cumulative tube length **(H)** of HUVECs with different treatments as indicated. **(I)** MTS assay was used to detect the proliferation of HUVECs. **(J)** Wound-healing assay was used to detect the migration of HUVECs; *N* = 5. Data are presented as mean ± SEM. *P < 0.05, Student’s *t*-test for **(A–E)** and ANOVA for **(F–J)**.

Next, we tested whether BMSCs-Exosomal miR-29a could regulate angiogenesis. First, miR-29a mimic was transfected into HUVECs to measure the effects of miR-29a on angiogenesis. MiR-29a level was dramatically increased in HUVECs transfected with miR-29a mimic compared to the control mimic group, indicating successful and effective transfection (Figure 3A). A tube-like structure formation assay showed that the cumulative tube length was significantly longer in miR-29a mimic-transfected HUVECs, in contrast to the control group (Figures 3B,C). As expected, HUVECs with the overexpression of miR-29a showed an enhanced ability of proliferation and migration in contrast to the control group (Figures 3D,E). Furthermore, BMSCs-Exos with miR-29a deficiency obtained from BMSCs with transfection of antagomir-29a were added into the culture medium of HUVECs. Consistently, BMSCs-Exos dramatically promoted tube-like structure formation, proliferation, and migration of HUVECs compared to those treated with the PBS control. However, the effects of BMSCs-Exos on angiogenesis were blunted with miR-29a deficiency in BMSCs-Exos (Figures 3F–J). These data indicated that miR-29a within exosomes played a key role in promoting angiogenesis by BMSCs-Exos.

### BMSCs-Exosomal miR-29a Regulated Angiogenesis *via* VASH1

**FIGURE 4:**
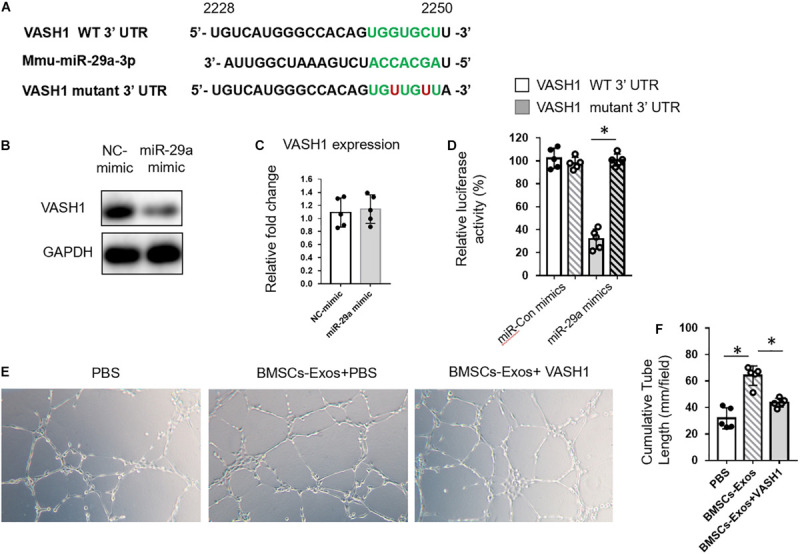
BMSCs-exosomal miR-29a regulated angiogenesis *via* VASH1. **(A)** The miR-29a targets were predicted using TargetScan. Western blot analysis **(B)** and qPCR analysis **(C)** of VASH1 in human umbilical vein endothelial cells (HUVECs) transfected with miR-29a or control mimics. **(D)** The impact of miR-29a mimics on WT-VASH1; the mutant-VASH1 luciferase activity in 293T cells is shown. Representative images of tube formation **(E)** and quantitative data of cumulative tube length **(F)** of HUVECs with different treatments as indicated; *N* = 5. Data are presented as mean ± SEM. *P < 0.05 as determined by Student’s *t*-test for **(C)** and ANOVA for **(D,F)**.

miRNAs bind to the amino acid coding sequence or 3′ UTRs of target genes to regulate gene expression. Target Scan was used to identify the targets of miR-29a. Among the predicted genes, we chose VASH1 for further study (Figure 4A and Supplementary Table S1). The reason that we chose VASH1 to study is that, among these target genes of miR-29a, VASH1 is a robust negative regulator of angiogenesis. Thus, we speculate that miR-29a might promote angiogenesis through post-transcriptional inhibition of VASH1. We have also listed the target genes that associated with osteogenesis and angiogenesis in Supplementary Table S1. The mRNA and protein levels of VASH1 were measured in HUVECs treated with miR-29a mimic or control by qPCR and western blotting analysis. miR-29a mimics downregulated VASH1 protein level but not mRNA level, indicating the post-transcriptional regulation of miR-29a on VASH1 gene (Figures 4B,C). A sequence analysis revealed that the binding site of miR-29a in the 3′UTR of VASH1 was from position 2244 to 2250 (Figure 4A). The direct binding of miR-29a on the seed region of VASH1 3′UTR was confirmed by luciferase reporter assay. Luciferase reporter constructs with wild-type 3′UTR of VASH1 (pGL3-VASH1^WT–3^′ UTR^) was generated and transfected into HEK293 cells with co-transfection of miR-29a mimics. The analysis of luciferase enzyme activity showed that miR-29a mimics significantly suppressed the luciferase activity of VASH1 3′UTR reporter gene. However, the mutation of two nucleotides in the putative binding sites in 3′UTR of VASH1 completely abolished these effects (Figure 4D). Furthermore, whether VASH1 mediates the effects of miR-29a on angiogenesis was tested. HUVECs transfected with miR-29a mimics were replenished with rVASH1, and a tube-like structure formation assay was conducted. Interestingly, the effect of miR-29a mimics on tube formation was blunted with the replenishment of rVASH1 (Figures 4E,F). These data indicated that VASH1, as a direct target, mediated the regulation of miR-29a on angiogenesis.

### BMSCs-miR-29a Exosomes Promoted Angiogenesis and Osteogenesis *in vivo*

**FIGURE 5:**
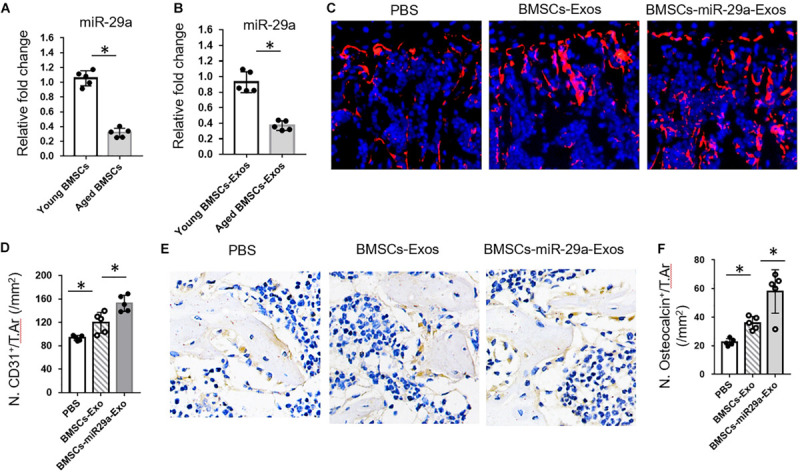
Bone marrow mesenchymal stem cells (BMSCs)-miR-29a exosomes promoted angiogenesis and osteogenesis. **(A**,**B)** qPCR analysis of miRNA-29a-3p expression in young (3 months) and aged (18 months) BMSCs and of exosomes isolated from young and aged BMSCs. The 3-month-old mice were injected with BMSCs-Exos, BMSCs-miR29a-Exos, or phosphate-buffered saline for 2 months. **(C)** Representative images of CD31 (red) immunostaining. Nuclei, 4′,6-diamidino-2-phenylindole (blue). **(D)** Quantitative analysis of CD31^+^ endothelial cells in distal femora. **(E)** Representative images of osteocalcin immunostaining. **(F)** Quantitative analysis of osteocalcin^+^ osteoblasts in distal femora (*N* = 5 mice in each group). Data are mean ± SD. *P < 0.05 as determined by ANOVA.

These above *in vitro* lead us to test the effects of BMSCs-Exos and miR-29a-loaded BMSCs-Exos on angiogenesis and osteogenesis *in vivo*. First, we isolated BMSCs-Exos from an age-related osteoporotic mouse model to measure the miR-29a level within exosomes. We found that the miR-29a level was dramatically decreased in aged BMSCs-Exo compared to young BMSCs-Exo, implying that the decreased level of miR-29a in aged exosome might contribute to age-related bone loss (Figures 5A,B). Next, BMSCs transfected with miR-29a mimics were subjected to collect the miR-29a-loaded BMSCs-Exos. The 3-month-old mice were treated with miR-29a-loaded BMSCs-Exos, BMSCs-Exos, or PBS control for 2 months. An immunostaining analysis revealed that the number of CD31-positive endothelial cells increased in mice treated with BMSCs-Exos compared to the PBS-treated control. More interestingly, mice with treatment of miR-29a-loaded BMSCs-Exos showed a greater number of endothelial cells than BMSCs-Exo-treated mice (Figures 5C,D) as angiogenesis is tightly coupled with osteogenesis. Next, we measured the effects of miR-29a-loaded BMSCs-Exos on osteogenesis. As expected, mice treated with miR-29a-loaded BMSCs-Exos have a greater number of osteocalcin-positive osteoblasts in bone surface compared with BMSCs-Exos- or PBS-treated mice (Figures 5E,F).

### miR-29a-Loaded BMSCs-Exos Increased Trabecular Bone Mass

**FIGURE 6:**
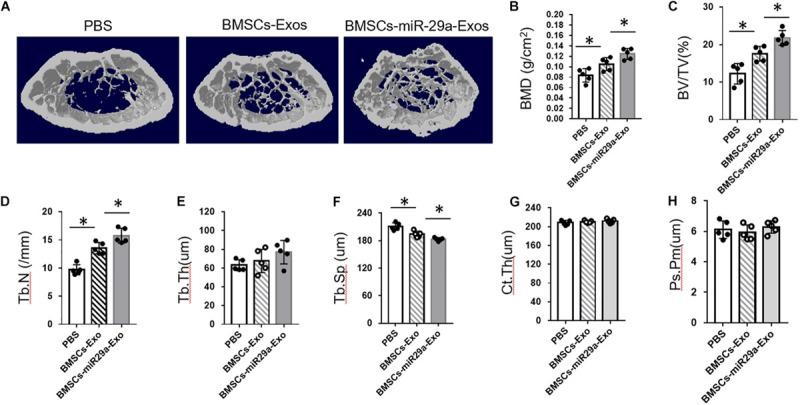
miR-29a-loaded bone marrow mesenchymal stem cells (BMSCs)-exosomes increased trabecular bone mass. The 3-month-old female mice were injected with BMSCs-Exos, BMSCs-miR29a-Exos, or phosphate-buffered saline for 2 months. Representative micro-CT images **(A)** and quantitative micro-CT analysis **(B**–**H)** of bone mineral density, trabecular bone volume (BV/TV), trabecular bone number (Tb.N), trabecular thickness (Tb. Th), trabecular separation (Tb. Sp), cortical thickness (Ct. Th), and periosteal perimeter (Ps. Pm) in femurs (*N* = 5 mice in each group). Data are mean ± SD (ANOVA). *P < 0.05 as determined by ANOVA.

Next, we tested the function of miR-29a-loaded BMSCs-Exos on bone mass. The 3-month-old mice were treated with miR-29a-loaded BMSCs-Exos, BMSCs-Exos, or PBS control for 2 months. A micro-CT analysis revealed that mice with treatment of BMSCs-Exos showed increased bone mineral density (BMD), trabecular bone volume (BV/TV), trabecular bone number (Tb.N), and lower trabecular separation (Tb. Sp) in contrast to the PBS-treated control. Moreover, the miR-29a-loaded BMSCs-Exos-treated mice showed additive elevation in BMD, BV/TV, and Tb.N when compared to BMSCs-Exos-treated mice (Figures 6A–F). We have also measured the effects of miR-29a-loaded BMSCs-Exos on cortical bone phenotype. However, we did not observe a significant change in cortical bone thickness in miR-29a-loaded BMSCs-Exos-treated mice compared to BMSCs-Exos- or PBS-treated mice (Figures 6G,H). These data indicate that miR-29a-loaded BMSCs-Exos have a robust ability of increasing bone mass in mice.

## Discussion

In this study, we have shown that BMSCs-Exos promote angiogenesis and osteogenesis. BMSCs-Exos can be taken up by endothelial cells and regulate angiogenesis. BMSCs-Exos promoted the tube-like structure formation, proliferation, and migration of HUVECs. We found that the miR-29a level was high in BMSCs-Exos and played key roles in regulating angiogenesis. Downregulation of miR-29a level in BMSCs-Exos showed blunted effects on promoting angiogenesis. Furthermore, we identified that VASH1, as a direct target of miR-29a, mediated the effects of BMSCs-Exos on angiogenesis. miR-29a-loaded BMSCs-Exos have a robust ability of increasing bone mass in mice. More interestingly, we found that miR-29a level was dramatically decreased in aged BMSCs-Exos compared to young BMSCs-Exos, implying that the decreased level of miR-29a in aged exosome might contribute to age-related bone loss, and miR-29a-loaded BMSCs-Exo might be a potential way to treat age-related osteoporosis. It is worthwhile to investigate the effect of miR-29a-loaded BMSCs-Exo in aged mice in a future study.

miR-29a belongs to miR-29 family which has been reported to regulate angiogenesis (Li Z. et al., 2017). miR-29a mimics regulate the angiogenesis of gastric carcinoma cells and downregulate VEGF level (Zhang et al., 2016). Besides the endogenous regulation, miR-29a/c can be secreted into microvesicles and transported into endothelial cells to affect angiogenesis. Zhang et al. (2016) found that upregulation of miR-29a/c level in microvesicles by transfection of miR-29a/c mimics in HEK293 cells suppresses VEGF expression and angiogenesis in gastric carcinoma cells. We found that BMSCs-exosomal miR-29a promoted angiogenesis. Although we found that miR-29a within BMSCs-Exos plays a vital role in regulating angiogenesis, we cannot exclude the possibilities that other microRNAs or small molecules within exosomes also contribute to the regulation of BMSCs-Exos on angiogenesis. Zheng et al. (2017) found that miR-155 mediates endothelial injury and atherosclerosis. MSC-derived exosomes promote endothelial cell angiogenesis by transferring miR-125a (Gong et al., 2017). Thus, screening other potential components in BMSCs-Exos regulating angiogenesis is worthy of future study.

Other than MSCs, other cell types, such as smooth muscle cells, adipose tissue macrophages, and tumor cells, also secret exosomes to mediate angiogenesis. Exosomal miR-155 from smooth muscle cells has been reported to induce endothelial injury and promote atherosclerosis (Zheng et al., 2017). Ovarian cancer cell-derived PKR1-positive exosomes promote migration and tube formation of HUVECs *in vitro* (Zhang et al., 2020). Adipocytes can secret exosomes to regulate angiogenesis in diabetic mice (Wang et al., 2018). The reason we chose BMSCs to study is that as functionally and numerically dominant in bone marrow, BMSCs show robust abilities of regulating angiogenesis and osteogenesis (Li et al., 2013, 2016a, 2018; Li C. et al., 2017). The effects of BMSCs-Exos and the key components within exosome on angiogenesis and osteogenesis in mice are not well studied. Thus, in this study, we tested the function of BMSCs-Exos on angiogenesis and osteogenesis.

Type H vessel has been reported to be coupled with osteogenesis in recent years. In this study, we found that miR-29a within BMSCs-Exos promoted angiogenesis. However, among the predicted target genes of miR-29a, there is no gene reported to be involved in the signaling or pathway associated with type H vessel formation. Thus, we did not test the effects of BMSC-derived exosomal miR-29a on type H vessel formation, whereas there is a possibility that miR-29a might regulate type H vessel formation through undefined signaling or pathway. It is worthwhile to test this hypothesis in a future study.

In our study, we observed increased bone mass in mice with the treatment of miR-29a-loaded BMSCs-Exos. In this study, we focus on the effects of miR-29a-loaded BMSCs-Exos on angiogenesis. We speculate that the increased angiogenesis may contribute to the increased bone mass in response to miR-29a-loaded BMSCs-Exos. However, miR-29a-loaded BMSCs-Exos may have direct effects on osteogenesis and/or osteoclastogenesis. Recent studies reported that microRNA-29a can repress osteoclastogenesis to counteract glucocorticoid-induced bone loss (Wu et al., 2019). Lian et al. (2019) found that miR-29a regulates PCAF-mediated RANKL and CXCL12 to repress osteoclast formation. MiR-29a has also been reported to regulate osteoblast differentiation of BMSCs in a high-fat environment through modulating Frizzled 4 (Liu F. et al., 2019). Thus, miR-29a-loaded BMSCs-Exos may have multiple effects on angiogenesis, osteogenesis, and osteoclastogenesis.

We did not observe a significant increase in cortical bone from mice treated with miR-29a-loaded BMSCs-Exos in contrast to those from PBS-treated mice. This may be because of the way and period of administration of exosomes. Previous studies reported that majority of exosomes accumulated in the liver and lung through tail vein injection as measured by fluorescence molecular tomography imaging system. Xie’s group reported that BMSC-specific aptamer conjugated exosomes have a high efficiency of bone tissue accumulation (Luo et al., 2019). Whereas they did not show the effects of BMSC-specific aptamer conjugated exosomes’ effect on cortical bone, the effects of bone tissue-targeted delivery of exosomes with a prolonged treatment time on cortical bone are an attractive topic for future study.

In this study, we found that miR29a-loaded BMSCs-Exos promote angiogenesis and osteogenesis. BMSCs-Exos promote the proliferation, migration, and tube formation of HUVECs. MiRNA-29a level was high in BMSCs-Exos and can be transported into HUVECs to regulate angiogenesis in a VASH1-dependent way. More interestingly, miR29a-loaded BMSCs-Exos have a robust ability of promoting angiogenesis and osteogenesis in mice. Thus, these findings suggest that BMSC-derived exosomal miR-29a regulates angiogenesis and osteogenesis, and miR-29a-loaded BMSCs-Exos may serve as a potential therapeutic target for osteoporosis.

## Data Availability Statement

The original contributions presented in the study are included in the article/Supplementary Material. Further inquiries can be directed to the corresponding author/s.

## Ethics Statement

Animal care and experimental protocol were approved by the Animal Care and Use Committees of the Laboratory Animal Research Center at Shihezi University First Affiliated Hospital.

## Author Contributions

ZW and TL designed the experiments, supervised the experiments, analyzed results, and wrote the manuscript. GL and PC carried out most of the experiments. All authors contributed to the article and approved the submitted version.

## Conflict of Interest

The authors declare that the research was conducted in the absence of any commercial or financial relationships that could be construed as a potential conflict of interest.
